# Promoting the Mental Health of University Students in China: Protocol for Contextual Assessment to Inform Intervention Design and Adaptation

**DOI:** 10.2196/25009

**Published:** 2021-05-11

**Authors:** Josephine Pui-Hing Wong, Cun-Xian Jia, Mandana Vahabi, Jenny Jing Wen Liu, Alan Tai-Wai Li, Xiaofeng Cong, Maurice Kwong-Lai Poon, Janet Yamada, Xuan Ning, Jianguo Gao, Shengli Cheng, Guoxiao Sun, Xinting Wang, Kenneth Po-Lun Fung

**Affiliations:** 1 Daphne Cockwell School of Nursing Ryerson University Toronto, ON Canada; 2 Department of Epidemiology, School of Public Health Cheeloo College of Medicine Shandong University Shandong China; 3 Psychiatry/Community Mental Health Toronto Western Hospital University Health Network Toronto, ON Canada; 4 Regent Park Community Health Centre Toronto, ON Canada; 5 Department of Social Work School of Political Science and Law University of Jinan Shandong China; 6 School of Social Work York University Toronto, ON Canada; 7 Department of Social Work School of Philosophy and Social Development Shandong University Shandong China; 8 School of Physical Education Shandong University Shandong China; 9 Department of Psychiatry Faculty of Medicine University of Toronto Toronto, ON Canada

**Keywords:** mental health, mental illness, stigma, protocol, acceptance and commitment therapy, implementation science, student mental health

## Abstract

**Background:**

Chinese students are extremely vulnerable to developing mental illness. The stigma associated with mental illness presents a barrier to seeking help for their mental health.

**Objective:**

The *Linking Hearts—Linking Youth and ‘Xin’ (hearts)* project is an implementation science project that seeks to reduce mental illness stigma and promote the mental health of university students in Jinan, China. The Linking Hearts project consists of 3 components. In this paper, we outline the protocol for the first component, that is, the contextual assessment and analysis of the mental health needs of university students as the first step to inform the adaptation of an evidence-based intervention to be implemented in Jinan, China.

**Methods:**

Six local universities will participate in the Linking Hearts project. A total of 100 students from each university (n=600) will engage in the contextual assessment through self-report surveys on depression, anxiety, stress, mental health knowledge, and mental health stigma. Quantitative data will be analyzed using several descriptive and inferential analyses via SPSS. A small number of participants (144 students and 144 service providers) will also be engaged in focus groups to assess the socio-environmental contexts of university students’ health and availability of mental health resources. Qualitative data will be transcribed verbatim and NVivo will be used for data management. Social network analysis will also be performed using EgoNet.

**Results:**

Linking Hearts was funded in January 2018 for 5 years. The protocol of Linking Hearts and its 3 components was approved by the research ethics boards of all participating institutions in China in November 2018. Canadian institutions that gave approval were Ryerson University (REB2018-455) in January 2019, University of Alberta (Pro00089364), York University (e2019-162) in May 2019, and University of Toronto (RIS37724) in August 2019. Data collection took place upon ethics approval and was completed in January 2020. A total of 600 students were surveyed. An additional 147 students and 138 service providers took part in focus groups. Data analysis is ongoing. Results will be published in 2021.

**Conclusions:**

Findings from this contextual assessment and analysis will generate new knowledge on university students’ mental health status, mental health knowledge, and resources available for them. These findings will be used to adapt and refine the *Acceptance and Commitment to Empowerment-Linking Youth N’ Xin* intervention model. The results of this contextual assessment will be used to inform the adaptation and refinement of the mental health intervention to promote the mental health of Chinese university students in Jinan.

**International Registered Report Identifier (IRRID):**

RR1-10.2196/25009

## Introduction

### Background

Young people constitute a particularly vulnerable population for the onset of major mental illness [1]. In China, the prevalence of depression and anxiety among children and youths aged 13-26 years ranges from 16% to 24% [2]. The prevalence of depression among Chinese university students is even higher, at approximately 20%-30% [3,4]. A meta-analysis of 10 cross-sectional studies, which together engaged over 30,000 Chinese medical students, reported the prevalence of depression at 29%, anxiety at 21%, suicidal ideation at 11%, and eating disorders at 2% [5]. These statistics suggest that university students in China are experiencing considerable mental health challenges. Academic pressure, achievement expectations, study stress, living away from home amidst many life changes, and ineffective coping strategies were found to be associated with mental distress and psychological disorders such as internet addiction, anxiety, and depression [2-4]. Targeted prevention, early identification, and intervention are critical in light of the increasing evidence on the association between the duration of untreated mental disorders and negative clinical outcomes [6]. In recent years, China has undertaken a significant service reform to expand mental health services beyond specialized hospitals. China’s second National Mental Health Work Plan (2015-2020) reconceptualizes mental health services in terms of prevention and promotion, treatment, and rehabilitation, and calls for integrated approaches of intergovernmental collaboration, active participation of community organizations, and proactive engagement of families and employers in promoting collective mental well-being [7]. The National Mental Health Work Plan emphasizes understanding, acceptance, and compassionate support for people living with mental illnesses, which is well aligned with the needs of university students. However, China’s existing mental health care systems are faced with numerous complex challenges that impede effective and responsive mental health promotion, prevention, and care. One of the major challenges is related to low or delayed to help-seeking among individuals and families affected by mental illness. Evidence shows that nearly 92% of the individuals diagnosed with mental disorders in China never sought mental health care [8-11]. Delayed help seeking is associated with the stigma of mental illness and low mental health literacy [12,13]. Further, evidence indicates that lay people in China, regardless of education levels, have limited understanding of mental illness and often assign causes of mental illness to individual personality traits and social skills [8]. In addition, mental health services, largely provided at specialized hospitals, are overstretched, and there exists a shortage of trained mental health professionals. All these factors pose tremendous challenges in the health care system to meet the population’s growing mental health needs [8]. One study found that 78% of the university health services are managed by student affair offices responsible for civil education and that mental health education is often marginalized [14]. Taken together, finding alternative strategies to meet Chinese university students’ mental health needs on campus and in the community is an important priority.

### Applying Evidence-Based Interventions in New Contexts

A number of evidence-based strategies have been used in the past to address system-level challenges, promote mental health, reduce stigma, and build capacity in individuals. One particular approach is the use of acceptance and commitment therapy (ACT), which is an evidence-informed, transdiagnostic intervention approach that is grounded on mindfulness [15]. ACT has previously been used with Chinese Canadian populations to reduce depression and adapted to promote mental health and reduce stigma for Asian men [16]. Given the congruency in the ACT processes of acceptance and value-oriented actions with Chinese cultural beliefs, ACT may be a useful intervention approach for adoption by the Chinese university population in Jinan, China.

Another approach that may be effective is group-based empowerment psychoeducation (GEP). This evidence-based approach is an adoption of traditional psychoeducation, whereby education extends beyond the provision of health information to include dialogue and critical reflection on the structural determinants of health and approaches that promote collective empowerment and capacity building among participants [17]. GEP has been successfully utilized in Chinese Canadians and other ethnoracial diasporic communities in previous studies [16]. GEP and ACT work in tandem to promote both individual and collective empowerment, and they reduce the stigma in Chinese university students in Jinan, China [11].

A growing body of evidence suggests that effective application and uptake of an evidence-based intervention must be informed by the contextual needs of the intended new users of the intervention [18,19]. The *Contextual Assessments and Analysis*, co-designed by the original intervention team and the knowledge user team, can provide critical information about the “who,” “what,” and “how” that are essential to ensure that the adapted intervention is relevant and effective to meet the sociocultural contexts of the intended end users and local stakeholders [20]. The use of mixed methods of quantitative and qualitative data collection and frequent dialogue enables the newly formed intervention team to identify the relevant needs for the users of the intervention, availability of resources and infrastructures, and acceptability of the intervention and implementation approaches [18].

### The Linking Hearts Project

The *Linking Hearts—Linking Youth and ‘Xin’ (hearts)* project is an implementation research undertaken by a Canada-China research partnership. The Linking Hearts team consists of over 30 interdisciplinary Canadian and Chinese researchers. This project is guided by a socio-ecological approach with the theoretical assumption that people’s experiences and health are influenced by factors at the individual, organizational, community, and societal levels. Since individuals shape and are shaped by their social contexts and environments, mental health challenges are too complex to be addressed from single-level analyses, and effective mental health interventions must consider multi-level contexts [21].

The objectives of Linking Hearts are to (1) improve access to quality mental health care for university students in Jinan, Shandong Province, China; (2) reduce stigma against mental illness that impedes help-seeking, targeted prevention, early identification, timely treatment, and optimal recovery; (3) improve interdisciplinary collaboration through collective empowerment and capacity building; and (4) advance implementation of science knowledge in the field of community mental health practices/interventions that can be scaled up in other real-life contexts. The overarching goal of Linking Hearts is to evaluate and document the process of intervention implementation and the resultant knowledge uptake by stakeholders and decision makers to transform existing professional capital in health and social care into expanded capacity in mental health care for university students. The processes of adaptation and implementation utilize an integrated, evidence-informed mental health intervention model, namely, acceptance and commitment to empowerment (ACE), which combines concepts and processes from ACT and GEP, and were found to be effective in reducing mental illness stigma and promoting mental health among Asians and other racialized groups in Canada [11]. Specifically, the team will adapt the ACE model into the *Acceptance and Commitment to Empowerment-Linking Youth N’ Xin* (ACE-LYNX) for use with university students.

### Three Components of the Linking Hearts Project

The Linking Hearts project consists of 3 components: (1) *contextual assessment and analysis* with service providers and students to inform the adaptation of the ACE intervention; (2) *implementation* of the refined ACE intervention with service providers and university students; and (3) *integrated knowledge translation* to engage diverse groups of knowledge users throughout all stages of this study. The Linking Hearts project activities are informed by the implementation science RE-AIM (Reach, Effectiveness, Adoption, Implementation, and Maintenance) framework of project planning that seeks to improve the sustainability of interventions by examining the reach, effectiveness, acceptability, implementation, and maintenance [22]. This paper describes the protocol of the first component of Linking Hearts, that is, the *contextual assessment and analysis* of mental health needs of Chinese students. In this component, we will engage service providers and university students to explore and identify the common mental health issues faced by students, their mental health literacy, and the contextual factors relevant to their mental health and resilience.

### Contextual Assessment of Mental Health Needs

As Linking Hearts is an international multi-interdisciplinary collaborative implementation research and the ACE intervention was developed and tested with Asian and racialized people in Canada, it is critical for the team to first conduct a contextual assessment and analysis of the needs of the students in Jinan. To effectively adapt ACE into the ACE-LYNX intervention for use with university students in Jinan, we will engage service providers and university students from 6 universities to identify the contexts and socio-environmental factors that determine the acceptability, feasibility, and effectiveness of ACE-LYNX. We will assess the psychological, cultural, social, and environmental contexts of university students in Jinan to gain a better understanding of their mental health needs in terms of not only challenges but also the resilience and protective factors of their mental health [23,24]. Knowledge generated from the contextual analysis will inform the adaptation of ACE into ACE-LYNX, a culturally relevant intervention.

## Methods

### Study Design

The conceptualization of the Linking Hearts Project was developed during a field visit by Canadian team members to Jinan. During this field visit, Canadian team members met with student groups, researchers, service providers at a community health center, and clinicians at the provincial mental health center. When the Linking Hearts Project was funded, the Jinan team also did a field visit to Toronto to meet with clinicians, on-campus counsellors, and community-based service providers to learn about community mental health. After these 2 field visits, the 2 teams held many teleconferences to develop the contextual assessment questions and data collection tools. The Linking Hearts Project was developed collaboratively between academic researchers and knowledge user researchers from Jinan, including counsellors on university campus and clinicians working in the mental health field. This study engages 2 participant populations—service providers and students. The researchers, professors, and service providers who are collaborators will be involved in the outreach and promotion to participants but not in direct recruitment.

### Data Collection Tools

The Linking Hearts project and study protocols have been approved by the research ethics boards of all participating institutions in China and Canada. Canadian institutions include Ryerson University (REB 2018-455), University of Toronto (RIS 37724), University of Alberta (Pro00089364), and York University (e2019-162). Informed consent will be obtained from all participants prior to data collection. The team will employ both qualitative and quantitative tools for data collection, including self-reported questionnaires, general response surveys, and focus groups conducted with students and service providers (Table 1).

**Table 1:** Design of the contextual assessment for each participating university site in Shandong, Jinan, China.

Participants			University 1		University 2		University 3		University 4		University 5		University 6
Students for quantitative survey (n=600)			100		100		100		100		100		100
**Sample for qualitative data and focus groups (n=288)**													
	Students	12		12		12		12		12		12	
	Service providers	12		12		12		12		12		12	

The survey and questionnaire instruments will include the following:

1. Student demographics: To better understand the social, economic, and cultural contexts of the university students whom we will engage in ACE-LYNX training, we will collect information on their background characteristics, program of study and year in program, living arrangement, family history, and family socioeconomic background.
2. Self-reported psychological symptoms: We will use the depression, anxiety, and stress scale (DASS-21), a widely used measure of self-reported mental health symptoms [25]. This measure contains 21 items, with 7 items in each of the 3 subscales intended to evaluate the depression, anxiety, and stress across student populations. Cronbach alpha coefficients of subscales with Chinese university student samples were acceptable, ranging from .72 to .81 in previous research [26]. The DASS-21 subscale totals will be interpreted along with the recommended cut-off scores for the interpretation of mild, moderate, and severe categorical distinctions of mental health symptoms.
3. Mental health literacy: Mental health knowledge and literacy will be evaluated using the mental health knowledge questionnaire (MHKQ) [27]. This measure consists of 20 true and false questions and 5 vignettes designed to evaluate mental health–related knowledge across general mental well-being issues [28]. The response rate of MHKQ in past research with Chinese samples ranged from 19% to 94%, with Cronbach alpha coefficients ranging from .57 to .73 [29]. The overall response rates, as well as individual question response rates, will be assessed in this study.
4. Mental illness stigma: The community attitudes toward mental illness (CAMI) scale is a 40-item questionnaire that measures externalized stigmatizing attitudes toward those with mental illness [30]. These include authoritarianism (viewing those with mental illness as inferior), benevolence (caring for the well-being of those mentally ill), social restrictiveness (seeing those with mental illness as a threat to society), and community ideology (accepting the therapeutic value and inclusion of mentally ill in society). The Cronbach alpha coefficients for subscales of the CAMI were reported to be acceptable, ranging from .60 to .81 in a sample of Asian men [16]. Subscale totals will be used as indicators of mental illness stigma in this study.

In addition to surveys and quantitative measures, participants will also be asked to identify their top mental health concerns, perceptions of the causes of mental illness, and areas of prioritization for service provision and training of health care professionals. We will list mental health resources based on information from the regional research team and advisory committees and ask participants to indicate whether they are aware of or have utilized them personally. In addition, participants will also be asked to list who they would turn to for advice regarding mental health issues. A small number of participants (144 university students and 144 service providers) will be recruited equitably from participating institutions by using stratified random sampling to participate in focus groups to explore (1) participants’ perspectives on mental health and mental illness, (2) common mental health needs of university students and the influencing factors, (3) how university students understand and respond to their mental health needs, (4) facilitators and barriers to accessing mental health care, (5) strategies to engage university students in mental health promotion and to improve access to care, and (6) participants’ perceived acceptability of the ACE-LYNX intervention to promote mental health among university students.

### Participant Populations and Inclusion Criteria

The target populations are health and social care providers and university students at 6 universities in Jinan, Shandong, China. The 6 universities were chosen based on (1) the diversity of the student population, that is, students from local and rural areas, (2) the diversity of disciplines and programs, and (3) their locations in each of the 3 regions within Shandong, China.

#### University Students

We will recruit 600 participants (100 per university site) to take part in a survey of general mental health-related symptoms, including depression, anxiety, and stress (DASS-21), stigma of mental illness (CAMI), mental health literacy (MHKQ), and mental health resources. Other measures evaluating clinical mental health symptomology were not included, as the purpose of the contextual analysis is to observe trends in mental health and resources supporting mental well-being. Inclusion criteria for participation include students, self-identified as 18 years of age or older, and attending 1 of the 6 partnering universities in Jinan (Table 1). They will also be asked complete the qualitative data collection tool to identify concerns and needs in mental health care. Survey participants interested in taking part in the follow-up focus group will be entered into a database. We will select 144 participants (24 per university site) to take part in focus groups to gain a deeper understanding of the complex contexts and factors of mental health issues among university students, including social structures and networks of access.

#### Psychiatric and Nonpsychiatric Health and Social Service Providers

At each university, we will host 2 focus groups (12 people each) with a total of 144 service providers to take part in a needs assessment. Inclusion criteria are service providers who self-identified as 18 years of age or older and provide health care, social care, or supportive services to university students in Jinan. Participants will first complete basic demographics information and information to identify concerns and needs in mental health care. Service providers participating will include psychiatrists, primary care physicians, nurses, social workers, university counsellors, and youth league leaders. Similar to above, focus groups will explore the complex contexts and factors of mental health issues among university students as well as identify training needs.

#### Participating and Collaborating Organizations

Partner organizations will keep track of and establish a 6-month baseline on quantities and types of services used by students. These organizations will include university counselling centers, individual school/faculty/departments at the 6 partner universities, community hospitals and clinics, and other regional organizations with stakes in the promotion of mental health and well-being of university students. These data will be used to measure reach and effectiveness.

### Data Analyses Plan

Quantitative data, including demographic information and descriptive statistics of the psychometric measures, will be tabulated from the student surveys by using SPSS (IBM Corp). Quantitative data will provide descriptive and inferential details regarding mental health trends and identify important resources for students and areas of needs regarding mental health support and service utilization. In addition, these mental health resources, including use of institutional programs and trusted advisors/consultants, will be collected, analyzed, and visualized with social network analysis methods using EgoNet (University of Florida). Qualitative data from focus groups will be transcribed verbatim, and NVivo (QSR International) will be used to aid in data management. We will use both inductive and deductive approaches to analyze the data. We will start with repeated readings of the transcripts to gain a broad understanding of the data. We will then perform line-by-line coding of key themes specific to the ideas articulated by the participants, followed by coding concepts/ideas posed in our research questions and derived from our theoretical understanding [31].

## Results

Linking Hearts was funded in January 2018 for 5 years. The protocol of Linking Hearts and its 3 components was approved by the research ethics boards of all participating institutions in China in November 2018. Canadian institutions that gave approval were Ryerson University (REB2018-455) in January 2019, University of Alberta (Pro00089364), York University (e2019-162) in May 2019, and University of Toronto (RIS37724) in August 2019. Data collection took place upon ethics approval and was completed in January 2020. A total of 600 students were surveyed. An additional 147 students and 138 service providers took part in focus groups. Data analysis is ongoing. Results will be published in 2021.

## Discussion

The results of the 3 types of data, that is, quantitative, qualitative, and social network, will form the basis of the contextual framework to guide the adaptation of the intervention to best suit the unique contexts and needs of Chinese university students in Jinan. Specifically, they will inform the adaptation of the psychoeducational component of the intervention (Mental Health 101). For example, aggregated survey results and relevant local statistics will be used to highlight the current trends of the mental health status and needs of university students. In addition, thematic analyses from the focus groups will inform the development of case examples used in the mental health education. Findings from the contextual assessment will be made available on the Linking Hearts project website.

## Abbreviations

ACE: acceptance and commitment to empowerment
ACE-LYNX: Acceptance and Commitment to Empowerment-Linking Youth N’ Xin
ACT: acceptance and commitment therapy
CAMI: community attitudes toward mental illness
DASS-21: depression, anxiety, and stress scale-21 item
GEP: group-based empowerment psychoeducation
MHKQ: mental health knowledge questionnaire
